# Impact of Polymorphic Variants on the Molecular Pharmacology of the Two-Agonist Conformations of the Human β1-Adrenoceptor

**DOI:** 10.1371/journal.pone.0077582

**Published:** 2013-11-08

**Authors:** Jillian G. Baker, Richard G. W. Proudman, Stephen J. Hill

**Affiliations:** Cell Signalling, School of Life Sciences, University of Nottingham, Nottingham, Nottinghamshire, United Kingdom; University of North Dakota, United States of America

## Abstract

β-blockers are widely used to improve symptoms and prolong life in heart disease primarily by inhibiting the actions of endogenous catecholamines at the β1-adrenoceptor. There are two common naturally occurring polymorphisms within the human β1-adrenoceptor sequence: Ser or Gly at position 49 in the N-terminus and Gly or Arg at position 389 in the C-terminus and some clinical studies have suggested that expression of certain variants may be associated with disease and affect response to treatment with β-blockers. The β1-adrenoceptor also exists in two agonist conformations - a high affinity catecholamine conformation and a low affinity secondary agonist conformation. Receptor-effector coupling and intracellular signalling from the different conformations may be affected by the polymorphic variants.

Here, we examine in detail the molecular pharmacology of the β1-adrenoceptor polymorphic variants with respect to ligand affinity, efficacy, activation of the different agonist conformations and signal transduction and determine whether the polymorphic variants do indeed affect this secondary conformation. Stable cell lines expressing the wildtype and polymorphic variants were constructed and receptor pharmacology examined using whole cell binding and intracellular secondary messenger techniques.

There was no difference in affinity for agonists and antagonists at the human wildtype β1-adrenoceptor (Ser49/Gly389) and the polymorphic variants Gly49/Gly389 and Ser49/Arg389. Furthermore, the polymorphic variant receptors both have two active agonist conformations with pharmacological properties similar to the wildtype receptor. Although the polymorphism at position 389 is thought to occur in an intracellular domain important for Gs-coupling, the two agonist conformations of the polymorphic variants stimulate intracellular signalling pathways, including Gs-cAMP intracellular signalling, in a manner very similar to that of the wildtype receptor.

## Introduction

β-blockers are widely used in the treatment of cardiovascular disorders and as well as improving symptoms (e.g. ischaemic heart disease and arrhythmias e.g. atrial fibrillation (AF)), they prolong life in patients with heart failure and post-myocardial infarction [1], [2]. The beneficial properties appear to be through antagonism of catecholamines at cardiac β-adrenoceptors (mainly β1-adrenoceptors) and the resulting reduction in rate and force of contraction improves both short and long term outcomes [2]–[5].

There are two common naturally occurring polymorphisms within the human β1-adrenoceptor; Serine (Ser) or Glycine (Gly) at position 49 in the N-terminus [6], [7] and Glycine (Gly) or Arginine (Arg) at position 389 in the C-terminus [6], [8], [9]. Although Ser49/Gly389 is known as the wildtype, the commonest variants are Ser49 found in 72–88% population and Arg389 present in 54–88% population (depending on ethnic origin, [1]).

At the cellular level, Gly49 β1-receptors appear to have a greater propensity to agonist-induced desensitization and downregulation than Ser49 receptors [10], [11] and thus Ser49 receptors have been called the “more active” variant [1]. In some clinical studies, Ser49 was associated with shorter survival in heart failure [7], [12], [13], increased risk of AF [14], and greater response to β-blockers [15], [16]. Arg389 β1-adrenoceptors appear better coupled to downstream Gs-cAMP signal transduction than Gly389 β1-adrenoceptors [8], [17]. Arg389 receptors have therefore been proposed to be the “more active” polymorphism. In some studies, this greater effector coupling of Arg389 receptors results in an increased heart rate, contractility and cardiac output in people in response to agonists compared to those with Gly389 [18], [19]. Arg389 individuals also have a greater response to β-blockers than Gly389 individuals [15], [20], [21]. In heart failure, some studies suggest that patients with Arg389 receptors have a longer survival [22] and a more beneficial response to β-blockers than those with Gly389 receptors [23]–[28]. In AF, patients with Gly389 receptors have a better response to β-blocker rate control than those with Arg389 receptors [29], [30] but ventricular arrhythmias appear to be more readily prevented in Arg389 than Gly389 patients [31].

However, there are also contrasting studies in the literature that did not find associations between genotype and disease outcome or response to treatment [1], [2], [5], [32]–[36]. There may be several explanations for this: the dose of β-blocker administered may not have been sufficient for the more active Ser49 and Arg389 receptor patients [12], [13], [37] or it may be due to the different β-blockers used in trials [15], [16], [20], [27], [30], [32].

At the molecular pharmacology level, the β1-adrenoceptor is known to exist in at least two agonist conformations: 1) a high affinity “catecholamine” conformation where agonist responses are readily inhibited by β-blockers and 2) a secondary low affinity agonist conformation where higher concentrations of β-blockers are needed to inhibit agonist responses [26], [38]–[41]. Interestingly, several clinically used β-blockers interact with this secondary conformation of the β1-adrenoceptor [42]–[46] and many β-blockers have been shown elicit agonist responses via this conformation [43]–[48].

A previous study has suggested that the coupling or signalling efficiency of this secondary conformation is affected by the polymorphic variant of the β1-adrenoceptor. The secondary conformation agonist CGP 12177 ((-)-4-(3-tert-butylamino-2-hydroxypropoxy)-benzimidazol-2-one) has been reported to be a very weak partial agonist at Arg389 receptors, whereas it was a full agonist at Gly389 receptors [17]. This suggests that the agonist efficacy of several clinically used β-blockers could vary between the different polymorphic variants of the human β1-adrenoceptor.

Here, we examine in detail the molecular pharmacology of the β1-adrenoceptor polymorphic variants with respect to ligand affinity and efficacy and we determine whether there are any differences in the molecular pharmacology between the catecholamine and secondary conformations of the wildtype and polymorphic variants of the human β1-adrenoceptor.

## Results

### ^3^H-CGP 12177 whole cell binding

**Figure 1:**
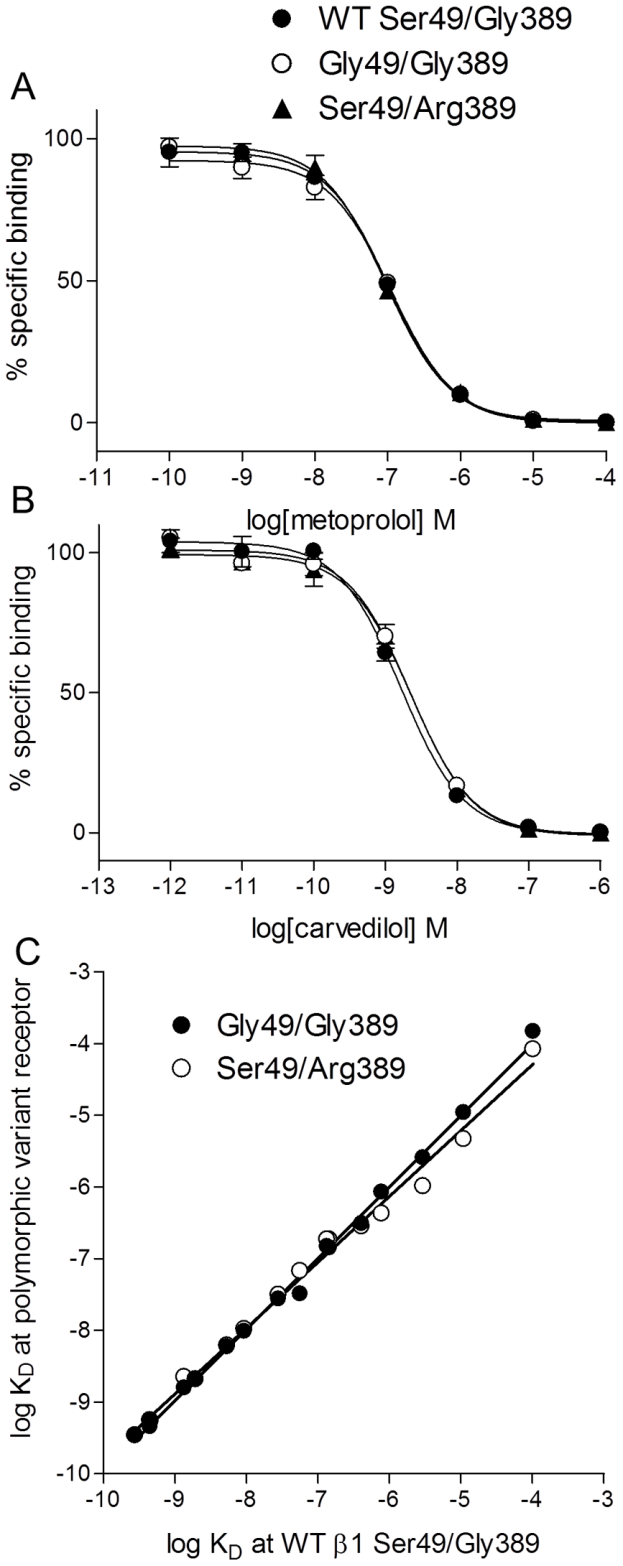
Inhibition of ^3^H-CGP 12177 binding at the wildtype receptor and polymorphic variants. Inhibition of ^3^H-CGP 12177 specific binding by **A** metoprolol and **B** carvedilol in wildtype (WT), Gly49/Gly389 cells and Ser49/Arg389 cells. Non-specific binding was determined by 10 µM propranolol. The concentration of ^3^H-CGP 12177 present in each case was 0.96 nM. Data points are mean ± s.e.mean of triplicate determinations and these single experiments are representative of 6 separate experiments in each case. **C** Correlation plot for the affinity of all the ligands from Table 1 for the wildtype (x-axis) and polymorphic variants (y-axis). There is a strong correlation between the affinity measurements made in the wildtype and those measured in either the Gly49/Gly389 receptor (R^2^ = 1.00, slope 0.99±0.01) and the Ser49/Arg389 receptor (R^2^ = 0.99, slope = 0.92±0.02). This demonstrates that ligands had very similar affinity for all three receptors.

Saturation binding revealed that ^3^H-CGP 12177 bound specifically to the wildtype β1-adrenoceptor (Ser49/Gly389) in the stable cell line with an affinity (K_D_ value) of 0.36±0.04 nM (receptor expression level 611±98 fmol/mg protein, n = 11). The affinity of ^3^H-CGP 12177 was similar in the β1Gly49/Gly389 cell line (K_D_ = 0.38±0.03 nM, 819±75 fmol/mg protein n = 10) and in the β1Ser49/Arg389 cells (K_D_ = 0.45±0.04 nM, 1068±142 fmol/mg protein n = 9). The binding affinities for several β-agonists and antagonists were then assessed by competition binding (Figure 1, Table 1).

**Table 1:** Affinity of β-adrenoceptor ligands for the wildtype and polymorphic variants of the β1-adrenoceptor.

	WT (Ser49/Gly389)	n	Gly49/Gly389	n	Ser49/Arg389	n
Adrenaline	−4.96±0.08	6	−4.96±0.04	6	−5.33±0.07##	6
Atenolol	−6.84 0.05	6	−6.85±0.05	6	−6.73±0.07	11
Bisoprolol	−8.03±0.05	5	−8.01±0.09	5	−7.98±0.04	5
Bucindolol	−9.56±0.05	6	−9.47±0.02	6	−9.46±0.03	6
Carvedilol	−9.34±0.03	6	−9.25±0.02	6	−9.26±0.05	6
CGP 20712A	−8.87±0.07	7	−8.80±0.07	7	−8.65±0.10	6
Cimaterol	−6.39±0.08	6	−6.51±0.09	5	−6.55±0.02	5
ICI 118551	−6.87±0.06	6	−6.83±0.03	6	−6.73±0.02	6
Isoprenaline	−6.11±0.07	6	−6.07±0.07	6	−6.37±0.10	3
Metoprolol	−7.55±0.02	6	−7.56±0.03	6	−7.50±0.04	6
Noradrenaline	−5.53±0.07	6	−5.59±0.04	6	−5.99±0.08###	6
Nebivolol	−9.35±0.06	6	−9.34±0.07	6	−9.25±0.07	6
Pindolol	−8.71±0.08	6	−8.67±0.08	6	−8.68±0.09	6
Propranolol	−8.27±0.04	6	−8.23±0.03	6	−8.21±0.03	11
Terbutaline	−3.99±0.04	6	−3.83±0.05#	6	−4.08±0.05	5
Xamoterol	−7.25±0.09	7	−7.19±0.03	7	−7.17±0.05	7

Log K_D_ values obtained from ^3^H-CGP 12177 whole cell binding in cells expressing either the human wildtype β1-adrenoceptor or the polymorphic variants. K_D_ values are mean ± s.e.mean for n separate experiments.

One-way ANOVA with post hoc Neuman-Keuls was performed comparing the affinities obtained for the wildtype receptor with those obtained for each polymorphic variant in turn.

# = p<0.05,

## = p<0.01,

### = p<0.001.

Statistically significant differences in affinities between the receptor polymorphisms were not seen in transiently transfected cells nor in all stable cell lines (Table S1 and S2 in File S1).

CGP 20712A (2-hydroxy-5-(2-[{hydroxy-3-(4-[1-methyl-4-trifluoromethyl-2-imidazolyl]phenoxy)propyl}amino]ethoxy)benzamide).

ICI 118551 ((-)-1-(2,3-[dihydro-7-methyl-1H-inden-4-yl]oxy)-3-([1-methylethyl]-amino)-2-butanol).

### ^3^H-cAMP accumulation

**Figure 2:**
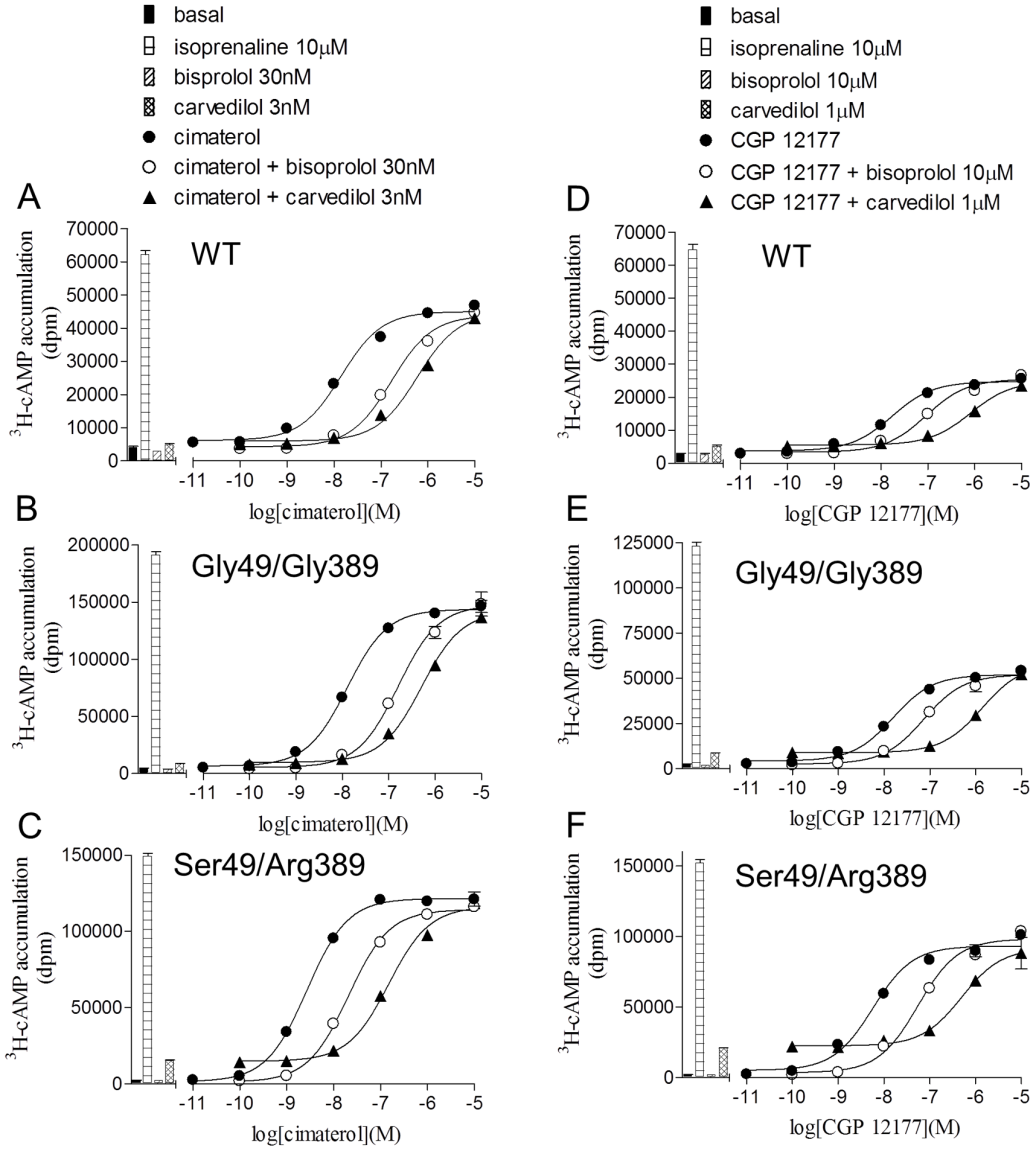
Inhibition of ^3^H-cAMP accumulation responses to cimaterol and CGP 12177 at the wildtype and polymorphic variant receptors. ^3^H-cAMP accumulation in response to cimaterol and CGP 12177 in **A** and **D** wildtype (WT) cells, **B** and **E** Gly49/Gly389 cells and **C** and **F** Ser49/Arg389 cells in the absence and presence of bisoprolol and carvedilol. Bars represent basal ^3^H-cAMP accumulation, that in response to 10 µM isoprenaline and that in response to 30 nM bisoprolol or 3 nM carvedilol for **A**, **B** and **C** or 10 µM bisoprolol or 1 µM carvedilol for **D**, **E** and **F**. Data points are mean ± s.e.mean of triplicate determinations and these single experiments are representative of 4 separate experiments in each case. This demonstrates the difference in the affinity of antagonists for the catecholamine (high affinity) conformation and secondary (low affinity) conformation of the receptors and that both of these conformations exist in all receptor variants.

Adrenaline and noradrenaline stimulated agonist responses at the wildtype β1-adrenoceptor and acted as full agonists relative to isoprenaline. Similar responses were seen in the Gly49/Gly389 and Ser49/Arg389 cell lines (Table 2). Cimaterol, an agonist known to stimulate responses via the high affinity catecholamine conformation of the wildtype receptor [45], stimulated slightly submaximal responses, and CGP 12177, a known secondary or low affinity conformation agonist, produced sub-maximal responses at all three receptors (Figure 2, Table 2). To confirm that these responses were occurring at the different conformations of the β1-adrenoceptor, they were inhibited by the β1-selective antagonist CGP 20712A and two common clinically used β-blockers, bisoprolol and carvedilol (Figure 2, Table 3). Inhibition of the cimaterol responses required low concentrations of β-blockers consistent with an interaction with the high affinity conformation, whereas inhibition of CGP 12177 responses required significantly higher concentrations of antagonist as required for the secondary low affinity conformation in all three cell lines.

**Table 2:** Agonist responses at the wildtype and polymorphic variants of the β1-adrenoceptor.

	WT Ser49/Gly389			Gly49/Gly389			Ser49/Arg389		
	Log EC_50_	% isop	n	Log EC_50_	% isop	n	Log EC_50_	% isop	n
Adrenaline	−6.68±0.26	96.4±2.1	5	−6.59±0.17	95.9±3.2	5	−6.92±0.24	91.6±2.2	5
Noradrenaline	−7.07±0.20	95.5±2.0	5	−7.00±0.25	99.1±3.7	5	−7.48±0.26	96.0±3.0	5
Cimaterol	−7.90±0.07	73.2±1.7	13	−7.91±0.03	77.4±1.1	15	−8.51±0.04#	87.1±1.6#	15
CGP 12177	−7.86±0.04	34.8±2.7	13	−7.69±0.03#	36.0±1.3	12	−8.11±0.08#	59.1±1.6#	13
CGP 20712A	No resp	0	4	No resp	0	4	No resp	0	4
Bisoprolol	No resp	0	4	No resp	0	4	No resp	0	4
Metoprolol	No resp	0	4	No resp	0	4	−7.66±0.10	0.7±0.1	4
Atenolol	No resp	0	4	No resp	0	4	−7.21±0.13	1.3±0.2	3
Nebivolol	−9.83±0.20	1.31±0.3	5	−9.42±0.09	1.16±0.1	5	−9.52±0.13	3.6±0.3#	5
Xamoterol	−8.13±0.06	28.0±2.4	7	−7.96±0.06	29.4±4.5	9	−8.05±0.10	52.1±4.5#	9

Log EC_50_ values and % isoprenaline maximal responses obtained from ^3^H-cAMP accumulation in cells expressing either the human wildtype β1-adrenoceptor or the polymorphic variants. Values are mean ± s.e.mean of n separate determinations.

One-way ANOVA with post hoc Neuman-Keuls was performed comparing the log EC_50_ values and % maximum isoprenaline responses obtained for the wildtype receptor with those obtained for each polymorphic variant in turn.

# = p<0.05.

The log EC_50_ values and % maximum isoprenaline responses for the partial agonists cimaterol, CGP 12177, nebivolol and xamoterol are significantly different for the Ser49/Arg389 receptor compared with wildtype. This could be due to either the higher receptor expression level of the Ser49/Arg389 cell line or more efficient receptor-effector coupling of the Ser49/Arg389 receptors (see Discussion for further detail).

**Table 3:** Affinity of antagonists for the two agonist conformations of the wildtype and polymorphic variants of the β1-adrenoceptor.

	K_D_ bisoprolol	n	K_D_ CGP 20712A	n	K_D_ carvedilol	n	K_D_ CGP 12177	n
Cimaterol as agonist								
WT Ser49/Gly389	−8.77±0.08	4	−9.85±0.07	7	−10.30±0.05	4	−9.90±0.07	8
Gly49/Gly389	−8.60±0.04	5	−9.83±0.08	14	−10.22±0.07	4	−9.90±0.05	8
Ser49/Arg389	−8.65±0.06	5	−9.78±0.03	13	−10.29±0.03	4	−9.83±0.05	6
CGP 12177 as agonist								
WT Ser49/Gly389	−5.72±0.04	5	−7.35±0.06	8	−7.55±0.08	5		
Gly49/Gly389	−5.70±0.06	5	−7.37±0.05	16	−7.73±0.14	5		
Ser49/Arg389	−6.04±0.12	5	−7.46±0.05	15	−7.93±0.10	5		

Log K_D_ values for bisoprolol, CGP 20712A, carvedilol and CGP 12177 obtained from ^3^H-cAMP accumulation the human wildtype β1-adrenoceptor or the polymorphic variant. Values are mean ± s.e.mean. of n separate determinations. The concentrations of antagonist use were: bisoprolol 30 nM when cimaterol was the agonist and 10 µM when CGP 12177 was the agonist; CGP 20712A 3 nM and 30 nM with cimaterol and 1 µM and 10 µM with CGP 12177; carvedilol 3 nM with cimaterol and 1 µM with CGP 12177; and CGP 12177 was at 1 nM and 10 nM when cimaterol was the agonist.

**Figure 3:**
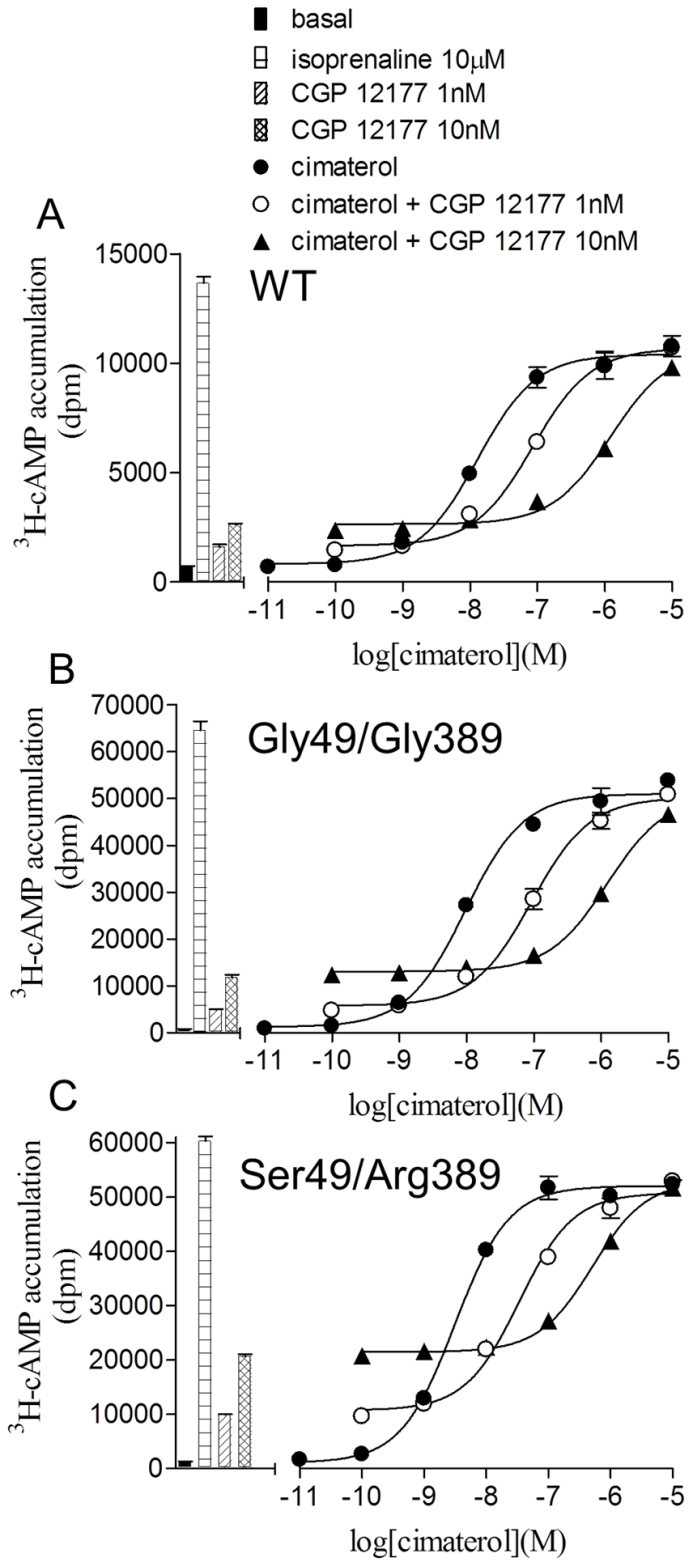
Inhibition of cimaterol-induced ^3^H-cAMP accumulation responses by CGP 12177. ^3^H-cAMP accumulation in response to cimaterol in **A** wildtype cells, **B** Gly49/Gly389 cells and **C** Ser49/Arg389 cells in the absence and presence of CGP 12177. Bars represent basal ^3^H-cAMP accumulation, that in response to 10 µM isoprenaline and that in response to 1 nM and 10 nM CGP 12177. Data points are mean ± s.e.mean of triplicate determinations and these single experiments are representative of 4 separate experiments in each case. Here, CGP 12177 inhibits the catecholamine conformation response with high affinity in all three receptor variants.

**Figure 4:**
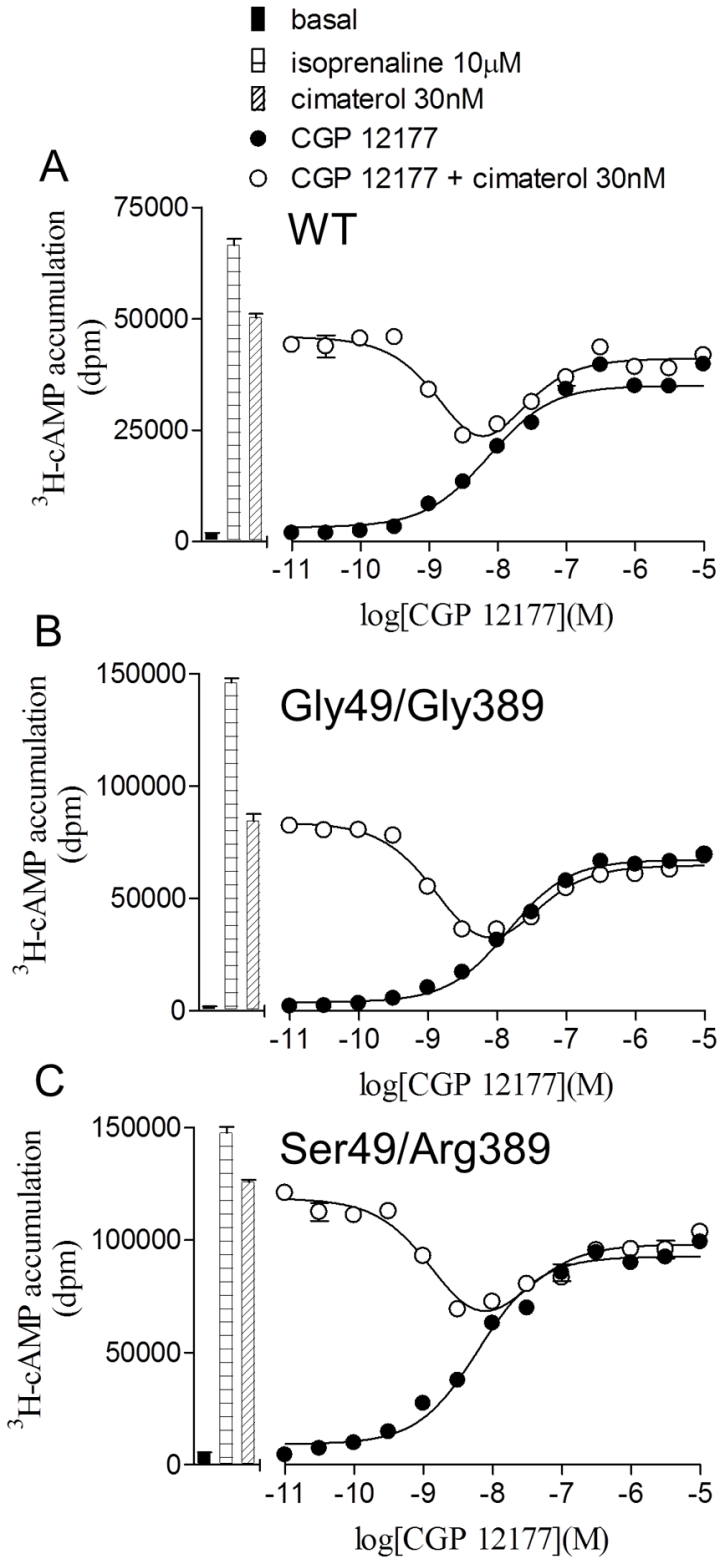
Demonstration of two agonist conformations of the wildtype β1-adrenoceptor and polymorphic variants. ^3^H-cAMP accumulation in response to CGP 12177 in **A** wildtype cells, **B** Gly49/Gly389 cells and **C** Ser49/Arg389 cells in the absence and presence of 30 nM cimaterol. Bars represent basal ^3^H-cAMP accumulation, that in response to 10 µM isoprenaline and that in response to 30 nM cimaterol. Data points are mean ± s.e.mean of triplicate determinations and these single experiments are representative of 5 separate experiments in each case. Low concentrations of CGP 12177 inhibit the high affinity catecholamine conformation and higher concentrations of CGP 12177 stimulate an agonist response. This demonstrates the two agonist conformations of the wildtype β1-adrenoceptor and that both of these agonist conformations are present in both polymorphic variants.

CGP 12177 is known to be a high affinity antagonist of the catecholamine conformation of the wildtype β1-adrenoceptor and inhibited the cimaterol response at very low concentrations to give high affinity K_D_ values for CGP 12177 (Figure 3, Table 3). High affinity antagonism of cimaterol can also be seen in Figure 4, where CGP 12177 (1–10 nM) inhibited the response to 30 nM cimaterol whereas 100 nM was required to achieve the maximum response to CGP 12177. A similar pattern was seen for both polymorphic variants of the receptor.

**Figure 5:**
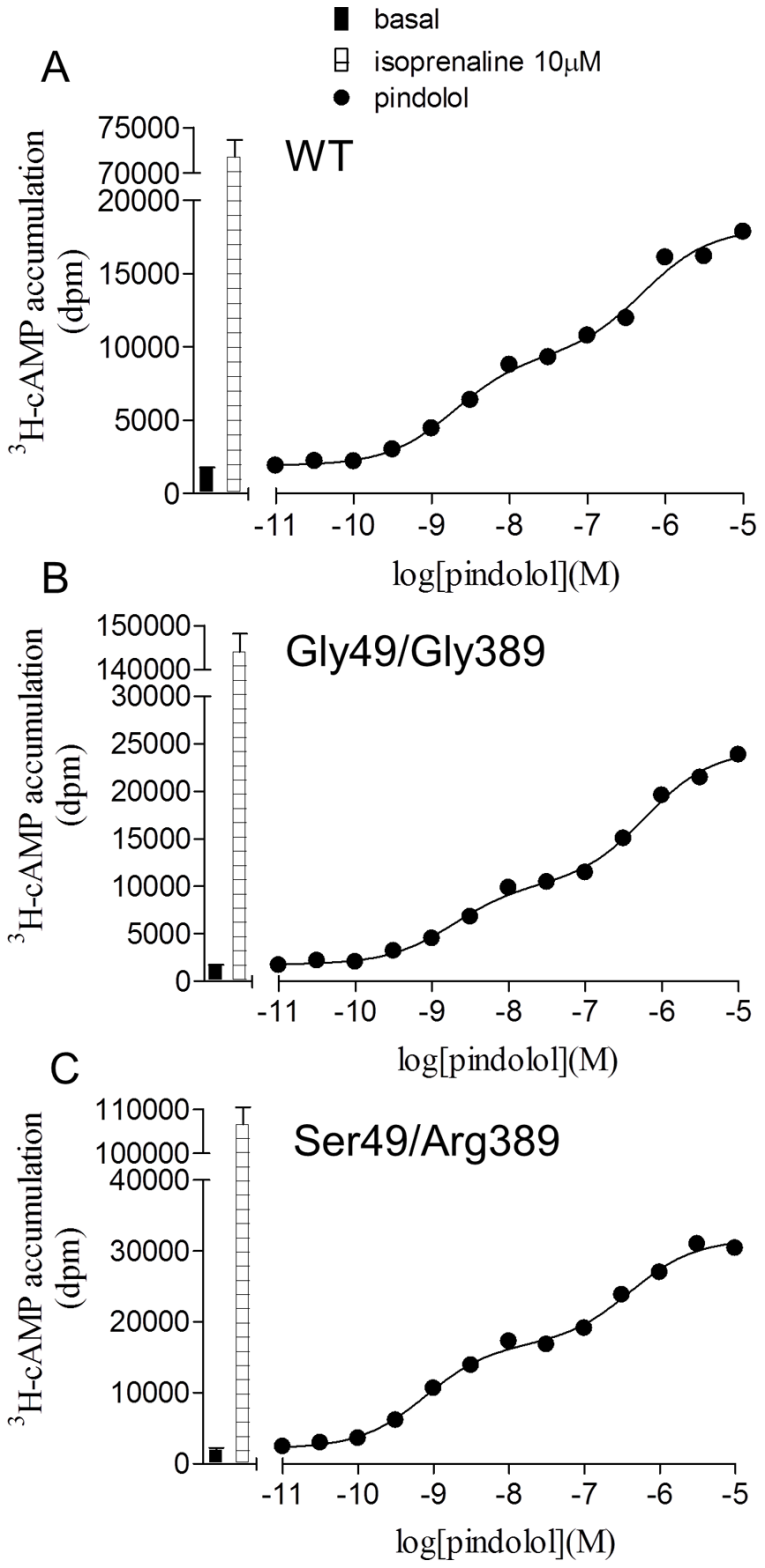
^3^H-cAMP accumulation in response to pindolol at the wildtype β1-adrenoceptor and polymorphic variants. ^3^H-cAMP accumulation in response to pindolol in **A** wildtype cells, **B** Gly49/Gly389 cells and **C** Ser49/Arg389 cells. Bars represent basal ^3^H-cAMP accumulation and that in response to 10 µM isoprenaline. Data points are mean ± s.e.mean of triplicate determinations. These single experiments are representative of 5 separate experiments in each case and demonstrate agonist actions at both agonist conformations of the β1-adrenoceptor in the wildtype and polymorphic variants.

The efficacy of other β-blockers used in clinical practice were then examined at the different polymorphic variants (Table 2). At the wildtype receptor, several ligands are known to stimulate both the high and low affinity conformations and yield biphasic concentration-response curves [43], [46], [53]. In the wildtype receptor, CGP 12177 produced a lower response than that mediated by the catecholamine conformation response to isoprenaline. However a previous pharmacological study found that at the secondary conformation of Arg389 receptors, CGP 12177 responses were very weak (5% that of the catecholamine conformation responses) whilst CGP 12177 responses were equally efficacious, stimulating the same size response as isoprenaline at Gly389 receptors [17]. A ligand with a biphasic concentration response in the wildtype receptor should therefore appear monophasic at the Arg389 receptor as the efficacy of ligands at the secondary conformation is so low as to be barely detectable. Here, however, similar biphasic responses were seen at the wildtype and both polymorphic variants of the receptor (Figure 5; Table 4).

**Table 4:** Agonist responses for ligands with biphasic responses at the wildtype and polymorphic variants of the β1-adrenoceptor.

	Log EC_50_ Site 1	Log EC_50_ Site 2	% Site 1	% isoprenaline	n
Pindolol					
WT Ser49/Gly389	−8.93±0.11	−6.17±0.24	45.6±3.7	14.5±2.3	5
Gly49/Gly389	−8.87±0.06	−6.03±0.11	42.2±2.6	13.7±0.9	5
Ser49/Arg389	−8.90±0.06	−6.13±0.08	50.4±3.9	34.6±1.7	5
Bucindolol					
WT Ser49/Gly389	−9.46±0.03	−7.53±0.05	59.8±2.8	24.6±4.1	5
Gly49/Gly389	−9.38±0.10	−7.45±0.11	51.5±2.4	25.7±0.8	5
Ser49/Arg389	−9.35±0.06	−6.98±0.19	80.4±2.6	53.6±1.2	5
Carvedilol					
WT Ser49/Gly389	−9.71±0.15	−7.23±0.10	52.1±5.7	5.8±1.1	8
Gly49/Gly389	−9.34±0.05	−7.37±0.12	47.2±1.8	4.3±0.5	9
Ser49/Arg389	−9.23±0.05	−7.24±0.07	54.2±2.3	17.8±4.8	8

Log EC_50_ values for ligands having a biphasic concentration response curve at the wildtype human β1-adrenoceptor and polymorphic variants. The % of the response occurring at site 1 and % maximum isoprenaline response for the overall response is also given. Values are mean ± s.e.mean of n separate determinations.

### Evidence of other G-protein coupling or signal transduction pathways

As the β1-adrenoceptor has been shown to have biased signalling [54], and the 389 polymorphism is in the region involved in G-protein coupling and signal transduction [55], the involvement of other signalling cascades was investigated.

**Figure 6:**
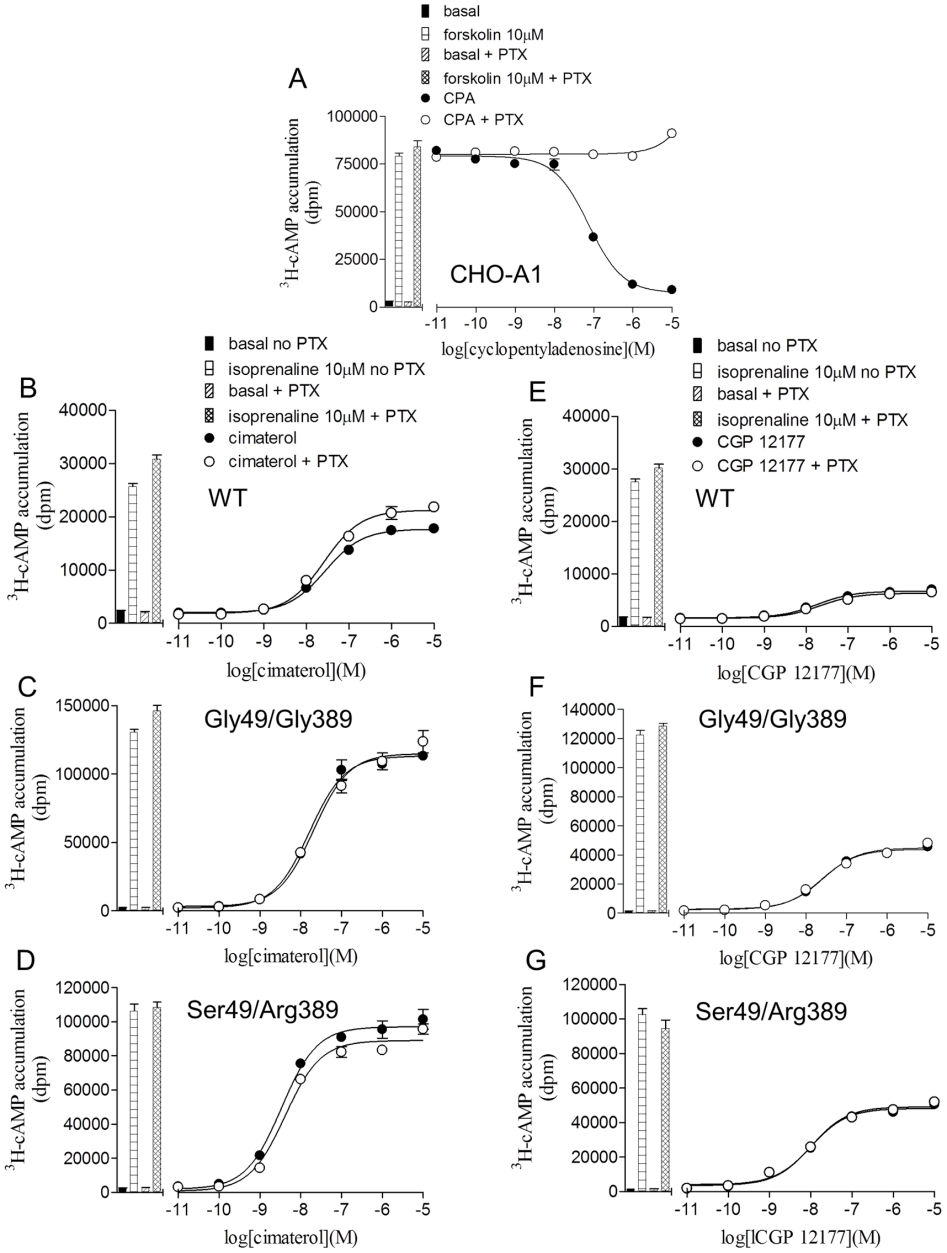
The effect of pre-incubation with pertussis toxin on ^3^H-cAMP accumulation responses. ^3^H-cAMP accumulation in **A** CHO cells expressing the human adenosine A1 receptor, **B** and **E** wildtype β1-adrenoceptor cells, **C** and **F** Gly49/Gly389 cells and **D** and **G** Ser49/Arg389 cells. All cells were subjected to 24 hours in serum free media before experimentations, those with closed circles in the absence of PTX and those with open circles in the presence of PTX. Bars represent **A** basal ^3^H-cAMP accumulation and that in response to 10 µM forskolin, **B–G** basal ^3^H-cAMP accumulation and that in response to 10 µM isoprenaline following incubation in serum free media without and with PTX. Data points are mean ± s.e.mean of triplicate determinations and these single experiments are representative of 4 separate experiments in each case. **A** demonstrates that the PTX pre-incubation was successfully preventing the Gi-coupled responses to cyclopentyladenosine (CPA) in CHO cells expressing the human A1 adenosine receptor, however there was no effect of PTX pre-incubation on the cimaterol (catecholamines conformation) or CGP 12177 (secondary conformation) responses occurring via the wildtype human β1-adrenoceptor or the polymorphic variants.

Pre-incubation with pertussis toxin (PTX) completely inhibited the Gi-coupled inhibition of ^3^H-cAMP accumulation in response to cyclopentyadenosine (CPA) in CHO cells expressing the human adenosine A1 receptor (Figure 6). However, it had no effect on the cimaterol or CGP 12177 responses at the WT, Gly49/Gly389 or Ser49/Arg389 receptors in experiments run on the β1-adrenoceptors in parallel experiments (Figure 6). In addition, PTX had no effect on the biphasic concentration response to pindolol (data not shown).

Signalling via the Gq pathways was examined using ^3^H-inositol phosphate accumulation. Here 100 µM UTP and 100 µM ATP stimulated responses via the endogenous P_2y2_ receptors [56] that were 1.87±0.11 and 2.18±0.17 n = 4 fold over basal at the wildtype Ser49/Gly389 receptor, 2.32±0.29 and 2.77±0.40, n = 4 at the Gly49/Gly389, and 2.63±0.40 and 2.96±0.28 n = 4 in the Ser49/Arg389 β1-receptors respectively. Even though the transfected β1-adrenoceptors were highly expressed, no agonist responses were seen to cimaterol (catecholamine conformation agonist), CGP 12177 (secondary conformation agonist), or pindolol (agonist at both conformations; Figure S1).

Finally, the potential for direct activation of the MAPKinase pathway was investigated using the Alphascreen Surefire MAPKinase assay. A time course was performed on all three receptors in case the maximum response occurred at different times for the two different agonist conformations of the receptor and for the positive control (phorbol ester). The maximum stimulation to 1 µM phorbol ester occurred at 30 minutes and was 11.9±1.6, 12.6±3.8 and 7.4±1.4, n = 3, fold over basal in the wildtype, Gly49/Gly389 and Ser49/Arg389 receptors respectively. Once again, although the transfected β1-adrenoceptors were highly expressed, no agonist responses were seen to cimaterol, CGP 12177, or pindolol (Figure S2).

### Impact of expression level - transient transfection and other cell lines

Using single stable cell lines to draw conclusions about receptor-effector coupling is difficult as each cell line is a unique entity – for example the place of insertion of the transfected DNA into the genome is unique in each line and may affect the expression of other genes as well as the expression of the receptor itself. As the main finding of this study differs from that previously published (with regard to the ability of the two agonist conformations to couple to downstream effectors) two further stable cells lines expressing either the wildtype receptor, Gly49/Gly389 or Ser49/Arg389 variants were also established. The responses obtained were very similar (see Tables S1, S3 and S4 in File S1, Figure S3). Also data similar to Figure 4 was obtained in all 9 cell lines. In addition, 3 or 4 separate transiently transfected populations of cells were examined for each of the wildtype receptor, Gly49/Gly389 or Ser49/Arg389 variants. Both variant receptors showed similar responses in terms of affinity (K_D_) and efficacy (EC_50_ and % maximum response) as the wildtype receptor (see Tables S2 and S3 in File S1). Thus similar data were found in 3 stable cell lines of the wildtype and each polymorphic variant and in 3–4 transiently transfected populations of each receptor.

## Discussion

Recent pharmacological and clinical studies have suggested that there are differences in cellular responses and clinical outcomes depending on which of the naturally occurring polymorphisms of the human β1-adrenoceptor are present. Here, we studied the wildtype human β1-adrenoceptor and its common polymorphic variants in detail, paying particular attention to the pharmacology of the two active conformations of the human β1-adrenoceptor - the high affinity catecholamine conformation and the low affinity secondary CGP 12177 conformation.

Firstly, the affinity with which agonists and antagonists bind to the wildtype receptor (Ser49/Gly389) and the two common polymorphic variants Gly49 and Arg389 was assessed by ^3^H-CGP 12177 whole cell binding. The affinity of ligands for the extracellular Ser49 and Gly49 polymorphic variants of the receptors [11] and the intracellular Gly389 and Arg389 variants [17] have previously been reported to be very similar. In keeping with these previous studies, the binding affinities of all ligands were found to be very similar across all three receptors variants (Figure 1, Table 1 and Figure S3, Table S1 and S2 in File S1 for other cell lines and transient transfections), suggesting that differences in affinity are not the explanation for the differences in clinical responses to β-blocker treatment seen in clinical trials.

The efficacy of ligands was then assessed by examining the primary signal transduction pathway: β1-Gs-coupled stimulation of cAMP. At the wildtype β1-adrenoceptor and both polymorphic variants, the endogenous catecholamine noradrenaline was more potent (left-shifted agonist concentration response) than adrenaline in keeping with expected β1-adrenoceptor activity (Table 2). When the responses to several partial agonists were examined (Table 2), it appears that the responses seen at the Ser49/Arg389 receptor were of greater efficacy (% maximum response and left-shifted EC_50_). This could be because of the higher receptor expression level in the Ser49/Arg389 cell line relative to the wildtype and Gly49/Gly389 cell lines or due to better coupling of the Ser49/Arg389 receptor to the Gs-cAMP effector system [8.17]. A change in the expression level of the wildtype receptor (from 219 to 2084 fmol/mg protein) causes a linear increase in the efficacy (% isoprenaline maximum response) of partial agonists (Table S5 in File S1) however this was not as clear in cell lines expressing the polymorphic variants where a smaller change in receptor expression level was achieved. It is therefore difficult to tease out whether the increased efficacy seen in the main Ser49/Arg389 cell line is indeed due to increased coupling efficiency or increased receptor expression level, or a combination of both. Transiently transfected studies however showed no change in partial agonist efficacy (see Table S3 in File S1). Whatever the reason, the increased efficacy unveils small agonist responses in this cell line, e.g. to atenolol and metoprolol, that are not visible at the other receptors.

The main aim of this study was to examine the pharmacological responses at the two agonist conformations of the β1-adrenoceptor. Cimaterol (a catecholamine, high affinity conformation agonist [45]) and CGP 12177 (a secondary, low affinity conformation agonist) both stimulated responses in the wildtype and both polymorphic variants (Table 2). However, whereas the cimaterol responses were antagonised by low concentrations of antagonists, much higher concentrations of antagonist were needed to antagonise the CGP 12177 response at all three receptors (Table 3 and Figure 2) in keeping with previous studies [17]. Secondly, low concentrations of CGP 12177 were able to inhibit the cimaterol response, thus leading to log K_D_ values of −9.90, −9.90 and −9.83 at the wildtype, Gly49/Gly389 and Ser49/Arg389 receptors respectively (Figure 3, Table 3) whereas the log EC_50_ values for the agonist responses stimulated by CGP 12177 were −7.86, −7.69 and −8.11. As the affinity (K_D_) and EC_50_ should be the same for a partial agonist interacting with a receptor at the same conformation, this suggests that CGP 12177 is acting through two different conformations of the receptor. Thirdly, CGP 12177 was able to inhibit the stimulatory response to cimaterol at concentrations lower than that required for it to stimulate an agonist response on its own (Figure 4). Taken together, therefore, this confirms that the two agonist conformations are present in both polymorphic variants as well as the wildtype β1-adrenoceptor.

Previous studies have, however, suggested that although both the high and low affinity conformations exist, they are coupled differently in the polymorphic variants. Joseph et al [17] found that whilst the isoprenaline response was greater in the Arg389 receptor compared to the Gly389 receptor (in keeping with greater Arg389 downstream coupling), CGP 12177 was a very weak partial agonist at the Arg389 receptor (5% response compared with isoprenaline) but stimulated full agonist response (105% compared with isoprenaline) at the Gly389 receptor. Also isoprenaline was found to be significantly more potent (EC_50_ value 100 times left-shifted) at the Gly389 receptor [17]. They therefore suggested that there were different modes of coupling for the catecholamine conformation and secondary conformation of the receptor.

Here, CGP 12177 stimulated agonist responses that were 34.8%, 36.0% and 59.1% of isoprenaline at the wildtype, Gly49/Gly389 and Ser49/Arg389 receptors respectively (Table 2, Figure 2), and thus were in proportion to that seen for other partial agonists (including xamoterol, previously been shown to be a catecholamine conformation partial agonist, [45]). This was also true for the other cell lines and the transient transfections (Table S3 and S4 in File S1). There is therefore no evidence here for differential secondary conformation coupling.

To examine this further, ligands that have agonist actions at both conformations of the β1-adrenoceptor (pindolol, carvedilol and bucindolol) were investigated (Table 4, Figure 5). These ligands have a biphasic concentration response where the first part of the response (low concentration) is thought to occur at the high affinity conformation as it is readily inhibited by antagonists while the second part of the response occurring at higher concentration is considered to be occurring at the low affinity secondary conformation of the receptor [43], [46], [53]. If the Arg389 primary conformation coupled well, and the secondary conformation very poorly, very little stimulation would be occurring via this secondary conformation and the concentration response curve should become monophasic. This was however not the case, and biphasic concentration response curves were seen at each polymorphic variant, for all the stable cell lines and the transient transfections (Table 4 and Table S4 in File S1). Furthermore, the proportions of the response occurring at each conformation was very similar across all the receptors. This once again suggests that the CGP 12177 secondary conformation couples to Gs-cAMP effectors in the wildtype and different polymorphic variants of the β1-adrenoceptor in a similar manner.

Finally, as the 389 polymorphism is within the intracellular Gs-coupling domain of the receptor and the Gly389 is thought to alter the α-helix of that region and therefore disrupt signalling [55], the ability of the wildtype and polymorphic variants to induce intracellular signalling via different mechanisms was examined, including other potential G-protein and non-G-protein coupled signalling [54], [57]–[59]. Pre-incubation with PTX, which prevents Gi/o-protein coupling by ADP ribosylating the Gαi/o subunit, had no effect on the cimaterol, CGP 12177 or biphasic pindolol responses, suggesting that coupling to Gi-proteins was not involved in the coupling of the catecholamine or secondary conformation responses in the wildtype or polymorphic variants. Similarly, cimaterol, CGP 12177 and pindolol did not stimulate an increase in ^3^H-inositol phosphate accumulation nor an increase in ERK1/2 MAPKinase activation. Therefore the polymorphic variants do not appear to differ in their ability to signal to other intracellular pathways.

In conclusion, this study suggests that there is no difference in affinity for agonists and antagonists at the human wildtype β1-adrenoceptor (Ser49/Gly389) and the polymorphic variants at positions Gly49/Gly389 and Ser49/Arg389. Furthermore, the polymorphic variant receptors both have two active agonist conformations with pharmacological properties the same as those of the wildtype receptor. Although the 389 polymorphism is thought to occur in an intracellular domain important for Gs-coupling, the two agonist conformations of the polymorphic variants stimulate intracellular signalling pathways, including Gs-cAMP intracellular signalling, in a manner very similar to that of the wildtype receptor.

## Materials and Methods

### Materials

Foetal calf serum which was from PAA Laboratories (Teddington, Middlesex, UK). Microscint 20 and Ultima Gold XR scintillation fluids were from PerkinElmer (Shelton, CT, USA). ^3^H-CGP 12177, ^3^H-adenine, ^14^C-cAMP and ^3^H-myo-inositol were from Amersham International (Buckinghamshire, UK). Bisoprolol, bucindolol, carvedilol, and cimaterol were from Tocris Life Sciences (Avonmouth, UK). Nebivolol was a gift from Stefano Evangelista (Menarini Ricerche Spa, Florence, Italy). The Surefire Alphascreen pERK1/2 kit was obtained from PerkinElmer. All other reagents were from Sigma Chemicals (Poole, Dorset, UK).

### Generation of human β1-adrenoceptor polymorphic variants

The cDNA sequence encoding the human β1-adrenoceptor in pJG3.6 was a gift from Steve Rees (GlaxoSmithKline, Stevenage,UK). This cDNA was subcloned as a HindIII/XbaI fragment into pcDNA3.1 (Invitrogen) and the sequence was confirmed by DNA sequencing. The wild-type (WT) β1-adrenoceptor sequence was confirmed to contain the Ser49 and Gly389 polymorphisms. Ser49Gly and Gly389Arg mutations were generated using the QuikChange mutagenesis kit (Stratagene, La Jolla, CA) and Bioline PolyMate Additive for GC-rich templates. Mutants are named as (wild-type residue)(residue number)(mutant residue). The sequence of the forward (F) and reverse (R) complementary oligonucleotide primers (5′ to 3′) used to synthesize a β1-adrenoceptor cDNA sequence containing the relevant mutations (indicated in bold) were as follows:

S49Gβ1-F CGCCAGCGAA**G**GCCCCGAGCCGC

S49Gβ1-R GCGGCTCGGGGC**C**TTCGCTGGCG

G389Rβ1-F CAAGGCCTTCCAG**C**GACTGCTCTGCTGCG

G389Rβ1-R CGCAGCAGAGCAGTC**G**CTGGAAGGCCTTG

After subcloning in Top F′ competent cells (Invitrogen), the mutant β1-adrenoceptor cDNA was excised on HindIII/XbaI and subcloned into native pcDNA3.1 containing a neomycin selection marker. All mutations and sequences were confirmed by DNA sequencing.

### Cell lines and cell culture

Chinese Hamster Ovary (CHO) cells were transfected with either the wildtype (WT) human β1-adrenoceptor (Ser49/Gly389) or each polymorphic variant in turn, either Gly49/Gly389 or Ser49/Arg389. The Gly49/Gly389 receptor rarely exists in humans [5]. The cells were selected for 3 weeks using resistance to geneticin (1 mg/ml) and stable cells lines were generated by dilution cloning. Where Gi-coupling was examined, CHO cells stably expressing the human adenosine A1 receptor (CHO-A1, [49]) were used as a control in each experiment to demonstrate that pertussis toxin (PTX) was indeed effective. All cells were grown in Dulbecco's modified Eagle's medium nutrient mix F12 (DMEM/F12) containing 10% foetal calf serum and 2 mM L-glutamine in a 37°C humidified 5% CO_2_ : 95% air atmosphere.

### ^3^H-CGP 12177 Whole Cell Binding

Cells were grown to confluence in white-sided tissue culture treated 96-well view plates and ^3^H-CGP 12177 whole cell saturation and competition binding was performed as previously described [46]. Briefly, 100 µl competing ligand in serum free media at twice the final required concentration was added to each well followed immediately by a 100 µl of ^3^H-CGP 12177 (giving a 1∶2 dilution in the well) and the plates incubated for 2 hours at 37°C, 5% CO_2_, humidified atmosphere. The cells were washed twice with 200 µl 4°C phosphate buffered saline, 100 µl Microscint 20 was added to each well, and the plates counted on a Topcount at 21°C for 2 minutes per well. Propranolol (10 µM) was used to define non-specific binding and ^3^H-CGP 12177 in the concentration range of 0.75–2.67 nM was used for competition assays and (0.005–46.7 nM) for saturation assays. Protein concentration was determined by the method of Lowry et al. [50].

### ^3^H-cAMP accumulation

Cells were grown to confluence in clear plastic tissue culture treated 24-well plates and ^3^H-cAMP accumulation performed as previously described [46]. Briefly, cells pre-labelled with ^3^H-adenine by incubation for 2 hours with 2 µCi/ml ^3^H-adenine in serum-free media (0.5 ml per well). The cells were washed, then 1 ml serum-free media containing 1 mM IBMX (3-isobutyl-1-methylxanthine) was added to each well and the cells incubated for 15 minutes. Agonists (in 10 µl serum-free media) were added to each well and the plates incubated for 5 hours in order to maximise the responses [46]. The assay was terminated by adding 50 µl concentrated HCl per well, the plates frozen, thawed and ^3^H-cAMP separated from other ^3^H-nucleotides by sequential Dowex and alumina column chromatography. Where CGP 12177 and cimaterol were co-incubated with the cells, the two ligands were added simultaneously. Where pertussis toxin (PTX) was used, cells were incubated in the 24 well plates with PTX at 100 ng/ml for 24 hours before experimentation. The response to cyclopentyladenosine (CPA) in the absence and presence of PTX was examined in CHO-A1 cells alongside every experiment to demonstrate that PTX was preventing Gi-coupled responses. 10 µM isoprenaline (or 10 µM forskolin in CHO-A1 cells) was used to define the maximal response in each experiment.

### ^3^H-inositol phosphate accumulation

Cells were grown to confluence in clear plastic tissue culture treated 24-well plates containing ^3^H-myo-inositol (4 µCi/ml). Cells were washed then incubated for 30 minutes in 1 ml of serum free media containing 20 mM LiCl (37°C, 5% CO_2_). Agonists (in 10 µl) were then added and the incubation continued for 1 hour. All reagents were then removed and 1 ml of cold (−20°C) methanol/0.12M HCl (1∶1, v/v) added to each well. The plates were stored at −20°C overnight before isolating total ^3^H-inositol phosphates as described previously [51]. Total ^3^H-inositol phosphate levels were determined by liquid scintillation counting. 100 µM ATP and 100 µM UTP was used as positive controls.

### MAPKinase - phospho ERK1/2

Extracellular-signal-regulated kinases ERK1/2 activation was measured using a Surefire Alphascreen pERK1/2 kit as per manufacturer's instructions. Briefly, cells were grown to confluence in clear plastic tissue culture treated 96-well plates then serum starved by incubation in serum-free media for 24 hours before experimentation. Agonist in 20 µl was added to the well (1∶5 dilution in wells) and incubated for 5–60 minutes (37°C, 5% CO_2_). Reagents were then removed and 20 µl lysis buffer added to each well. Plates were frozen at −20°C overnight, thawed, and the assay then conducted as per manufacturer's instructions. After 2 hours in the dark, the plates were read on an Envision plate reader using standard Alphascreen settings. 1 µM phorbol ester was used as the positive control.

### Data analysis

#### Whole cell binding - Saturation binding

To determine the binding affinity (K_D_ value) for ^3^H-CGP 12177 and the receptor expression level, saturation binding curves of the total and non-specific binding (as determined by the presence of 10 µM propranolol) were performed with all data points in quadruplicate. Specific binding (SB, equation 1) of ^3^H-CGP 12177 at different concentrations of the ^3^H-ligand was plotted using the non-linear regression program Prism 2 to the equation:
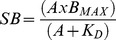
(1)where A is the concentration of ^3^H-CGP 12177, B_MAX_ is the maximal specific binding and K_D_ is the dissociation constant of ^3^H-CGP 12177.

#### Whole cell binding - Competition binding

In all cases, the competing ligand completely inhibited the specific binding of ^3^H-CGP 12177. All data points were performed in triplicate and each 96-well plate also contained 6 determinations of total and non-specific binding. A one-site sigmoidal response curve was then fitted to the data using Graphpad Prism 2.01 and the IC_50_ was then determined as the concentration required to inhibit 50% of the specific binding.
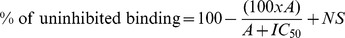
(2)where A in the concentration of the competing ligand, IC_50_ is the concentration at which half of the specific binding of ^3^H-CGP 12177 has been inhibited, and NS is the non-specific binding.

From the IC_50_ value and concentration of radioligand [^3^H-CGP 12177], the K_D_ value (concentration at which half the receptors are bound by the competing ligand) was calculated using the equation:
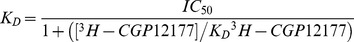
(3)


#### Functional assays - 3H-cAMP accumulation

Most agonist responses were best described by a one-site sigmoidal concentration response curve (equation 4)
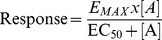
(4)Where Emax is the maximum response, [A] is the agonist concentration and EC_50_ is the concentration of agonist that produces 50% of the maximal response

The affinities of antagonists (K_D_ values, Table 3) were calculated from the shift of the agonist concentration response curve in the presence of a fixed concentration of antagonist using equation 5:
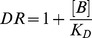
(5)where DR (dose ratio) is the ratio of the agonist concentration required to stimulate an identical response in the presence and absence of a fixed concentration of antagonist [B].

When CGP 12177 was used as an antagonist, clear partial agonism was seen (Figure 3). Here, the affinity was calculated by the method of Stephenson [52] using equation 6:
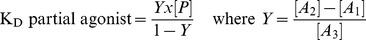
(6)where [P] is the concentration of CGP 12177, [A_1_] is the concentration of cimaterol at the point where CGP 12177 alone causes the same response, [A_2_] is the concentration of cimaterol causing a given response above that achieved by CGP 12177 and [A_3_] the concentration of cimaterol, in the presence CGP 12177, causing the same stimulation as [A_2_].

Several of the responses were, however, best described by a two-site concentration response using equation 7 (e.g. Figure 5, Table 4)
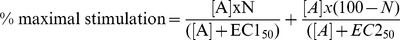
(7)where N is the percentage of site 1, [A] is the concentration of agonist and EC1_50_ and EC2_50_ are the respective EC_50_ values for the two agonist sites.

A two-site analysis was also used for the experiments shown in Figure 4 using equation 8:
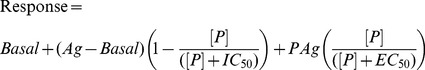
(8)where basal is the response in the absence of cimaterol, Ag is the response to a fixed concentration of cimaterol, [P] is the concentration of the partial agonist CGP 12177, IC_50_ is the concentration of CGP 12177 that inhibits 50% of the response to cimaterol, PAg is the maximum stimulation by CGP 12177 and EC_50_ is the concentration of CGP 12177 that stimulated a half maximal CGP 12177 response.

All data are presented as mean ± s.e.m. of triplicate determinations (except saturation binding experiments where determinations were from quadruplicate wells) and n in the table and text refers to the number of separate experiments.

## Supporting Information

Figure S1 **^3^H-inositol phosphate accumulation in cells expressing the wildtype β1-adrenoceptor and polymorphic variants.** ^3^H-inositol phosphate accumulation in A wildtype cells, B Gly49/Gly389 cells and C Ser49/Arg389 cells. Bars are mean ± s.e.mean of triplicate determinations. These single experiments are representative of 4 separate experiments in each case and demonstrate a lack of Gq-coupled inositol phosphate accumulation in response to β-adrenoceptor ligands.
(TIF)

Figure S2 **MAPKinase activation of the wildtype β1-adrenoceptor and polymorphic variants.** MAPKinase activation in A wildtype cells, B Gly49/Gly389 cells and C Ser49/Arg389 cells. Bars are mean ± s.e.mean of triplicate determinations. These single experiments are representative of 3 separate experiments in each case and demonstrate a lack of ERK1/2 MAPKinase stimulation by β-adrenoceptor ligands.
(TIF)

Figure S3 **Correlation plot and statistical analysis for the affinity of all the ligands from Table S1 in File S1 for the WT (clone T88 x-axis) and WT (clone T3), polymorphic variants, and transient populations (y-axis).** The linear regression lines are not shown on the graph to ensure that the symbols are still visible. This shows that the affinity of ligands for the different β1-adrenoceptor variants is similar to that for the wildtype receptor.
(TIF)

File S1 **Table S1, Affinity of β-adrenoceptor ligands for the wildtype and polymorphic variants of the β1-adrenoceptor. Table S2, Affinity of β-adrenoceptor ligands for the wildtype and polymorphic variants of the β1-adrenoceptor in transiently transfected populations. Table S3, Agonist responses occurring via the wildtype and polymorphic variants of the β1-adrenoceptor. Table S4, Agonist responses for ligands with biphasic responses at the wildtype and polymorphic variants of the β1-adrenoceptor in two additional stable cell lines for each receptor and transiently transfected cells. Table S5, Relationship between receptor expression level and efficacy of partial agonists at the two agonist conformations of the wildtype and polymorphic variants of the β1-adrenoceptor.**
(DOC)
