# Supplementary figures and images for: Impact of Polymorphic Variants on the Molecular Pharmacology of the Two-Agonist Conformations of the Human β1-Adrenoceptor
Source: PLoS One. 2013 Nov 8;8(11):e77582. doi: 10.1371/journal.pone.0077582 (PMC3826719; doi:10.1371/journal.pone.0077582)

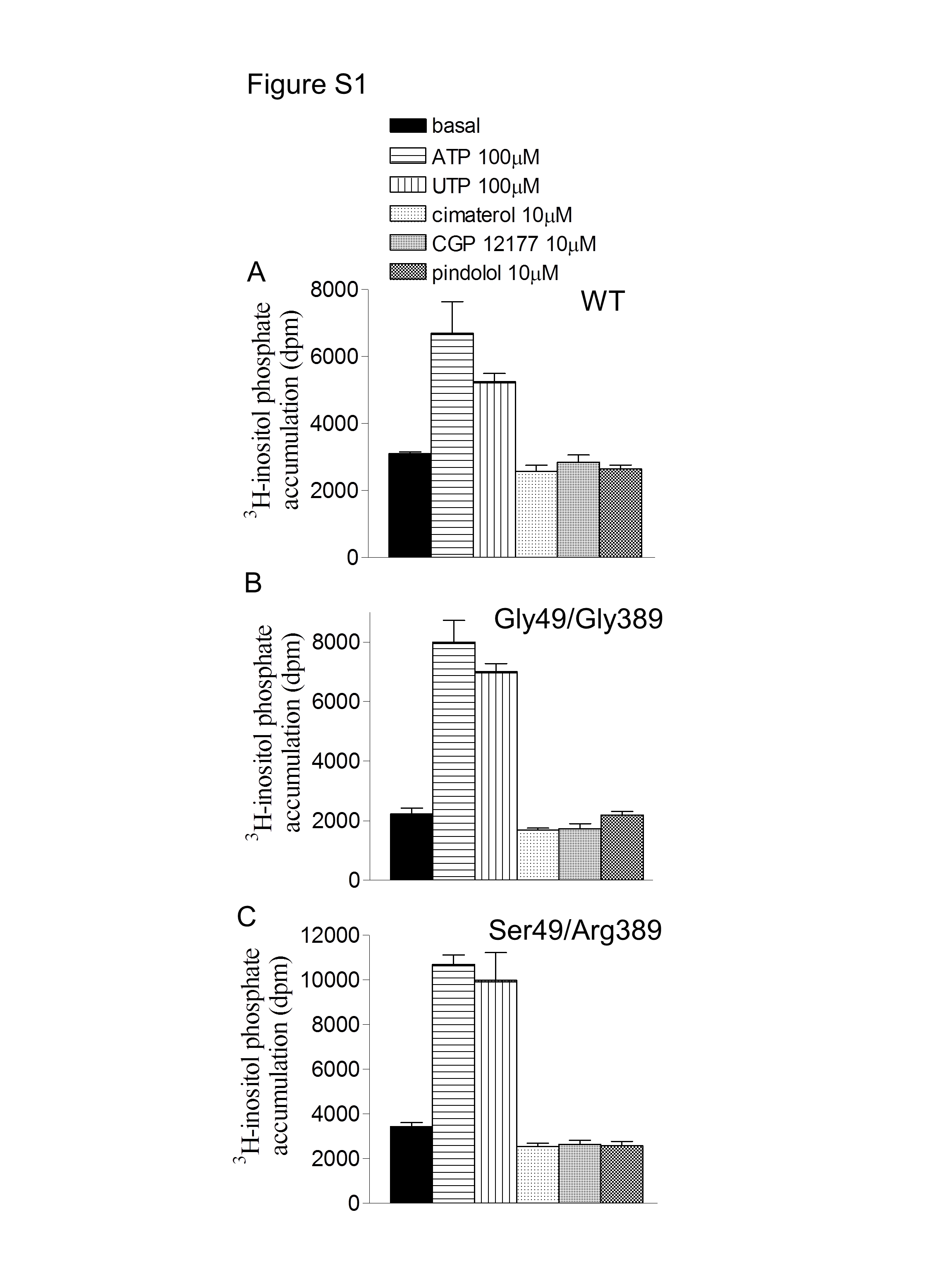

Supplement: Figure S1 — 3H-inositol phosphate accumulation in cells expressing the wildtype β1-adrenoceptor and polymorphic variants. 3H-inositol phosphate accumulation in A wildtype cells, B Gly49/Gly389 cells and C Ser49/Arg389 cells. Bars are mean ± s.e.mean of triplicate determinations. These single experiments are representative of 4 separate experiments in each case and demonstrate a lack of Gq-coupled inositol phosphate accumulation in response to β-adrenoceptor ligands. (TIF) [file pone.0077582.s001.tif]

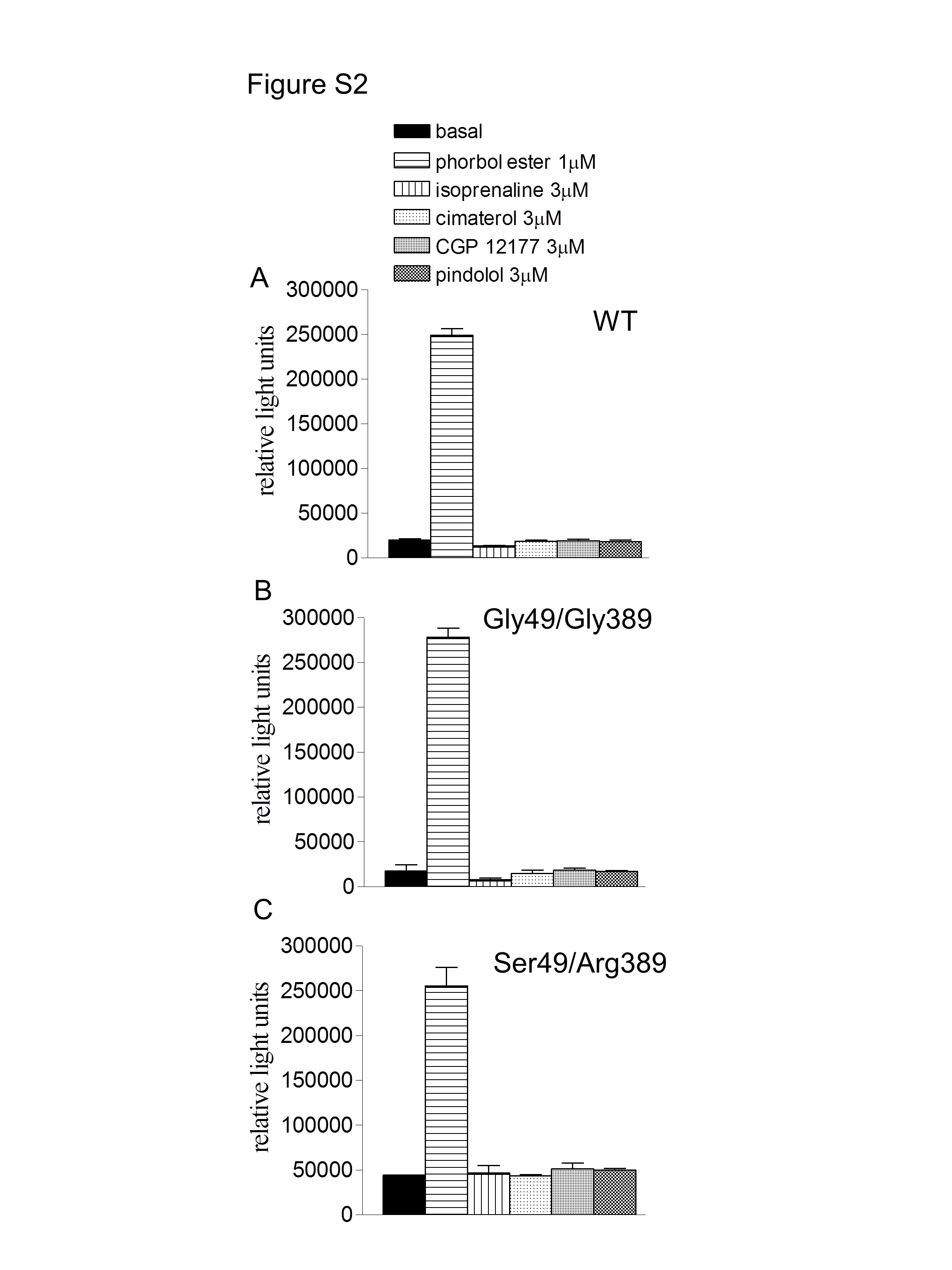

Supplement: Figure S2 — MAPKinase activation of the wildtype β1-adrenoceptor and polymorphic variants. MAPKinase activation in A wildtype cells, B Gly49/Gly389 cells and C Ser49/Arg389 cells. Bars are mean ± s.e.mean of triplicate determinations. These single experiments are representative of 3 separate experiments in each case and demonstrate a lack of ERK1/2 MAPKinase stimulation by β-adrenoceptor ligands. (TIF) [file pone.0077582.s002.tif]

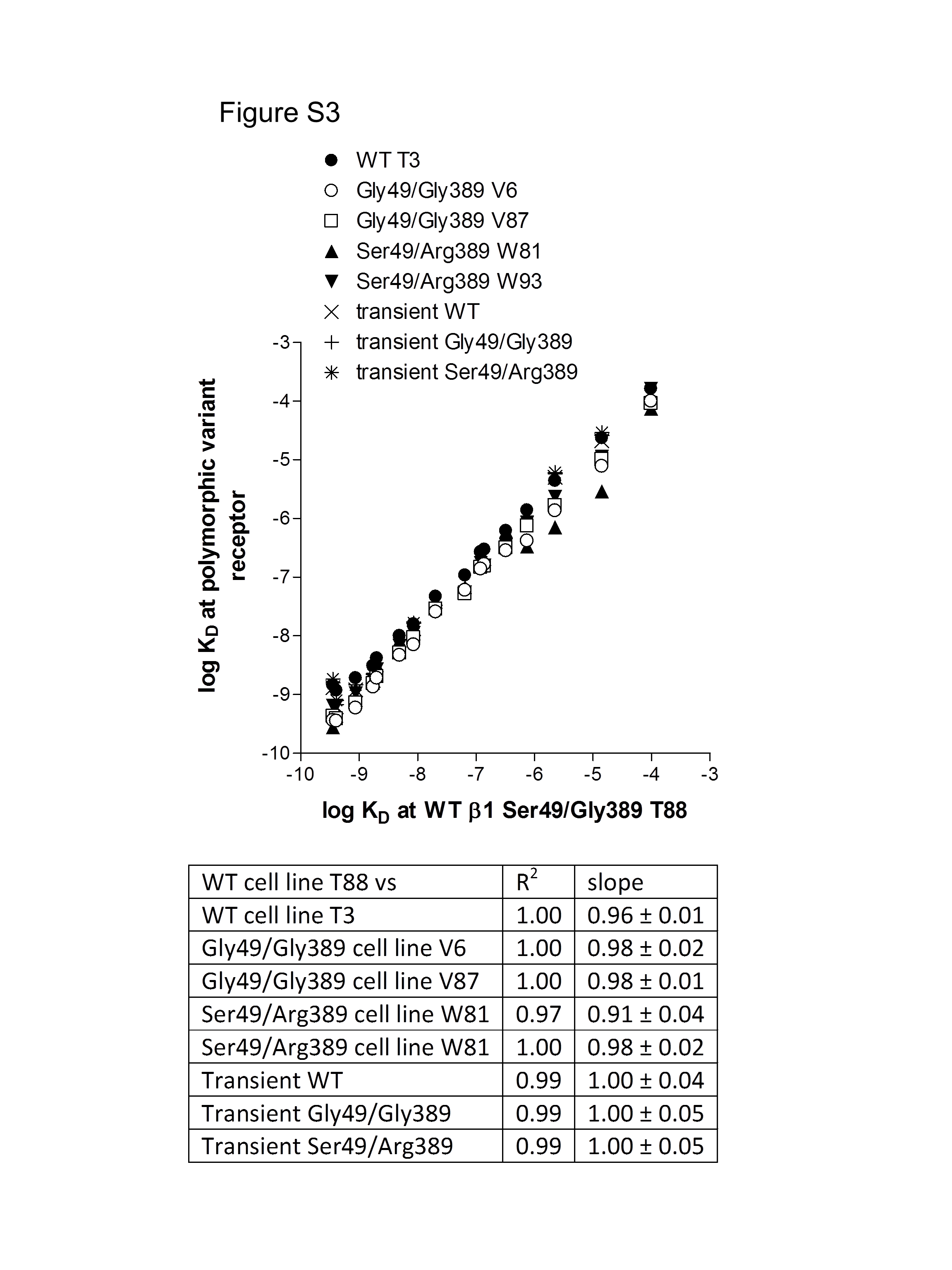

Supplement: Figure S3 — Correlation plot and statistical analysis for the affinity of all the ligands from Table S1 in File S1 for the WT (clone T88 x-axis) and WT (clone T3), polymorphic variants, and transient populations (y-axis). The linear regression lines are not shown on the graph to ensure that the symbols are still visible. This shows that the affinity of ligands for the different β1-adrenoceptor variants is similar to that for the wildtype receptor. (TIF) [file pone.0077582.s003.tif]
