# Supplementary material for: Impact of Polymorphic Variants on the Molecular Pharmacology of the Two-Agonist Conformations of the Human β1-Adrenoceptor
Source: PLoS One. 2013 Nov 8;8(11):e77582. doi: 10.1371/journal.pone.0077582 (PMC3826719; doi:10.1371/journal.pone.0077582)
Supplement: File S1 — Table S1, Affinity of β-adrenoceptor ligands for the wildtype and polymorphic variants of the β1-adrenoceptor. Table S2, Affinity of β-adrenoceptor ligands for the wildtype and polymorphic variants of the β1-adrenoceptor in transiently transfected populations. Table S3, Agonist responses occurring via the wildtype and polymorphic variants of the β1-adrenoceptor. Table S4, Agonist responses for ligands with biphasic responses at the wildtype and polymorphic variants of the β1-adrenoceptor in two additional stable cell lines for each receptor and transiently transfected cells. Table S5, Relationship between receptor expression level and efficacy of partial agonists at the two agonist conformations of the wildtype and polymorphic variants of the β1-adrenoceptor. (DOC) [file pone.0077582.s004.doc]

Table S1.

Affinity of β-adrenoceptor ligands for the wildtype and polymorphic variants of the β1-adrenoceptor

|  | WT | | | | Gly49/Gly389 | | | | Ser49/Arg389 | | | |
| --- | --- | --- | --- | --- | --- | --- | --- | --- | --- | --- | --- | --- |
|  | cell line T3 | n | cell line T88 | n | Cell line V6 | n | Cell line V87 | n | Cell line W81 | n | Cell line W93 | n |
| Adrenaline | -4.63 ± 0.04 | 5 | -4.85 ± 0.05 | 5 | -5.11 ± 0.06# | 5 | -4.99 ± 0.02 | 5 | -5.54 ± 0.04## | 5 | -4.95 ± 0.09 | 5 |
| Atenolol | -6.53 ± 0.06 | 8 | -6.86 ± 0.13 | 8 | -6.78 ± 0.07 | 8 | -6.81 ± 0.07 | 8 | -6.69 ± 0.09 | 8 | -6.80 ± 0.04 | 3 |
| Bisoprolol | -7.80 ± 0.04 | 6 | -8.07 ± 0.05 | 6 | -8.15 ± 0.04 | 6 | -8.03 ± 0.05 | 6 | -7.96 ± 0.02 | 6 | -7.95 ± 0.05 | 6 |
| Bucindolol | -8.84 ± 0.01 | 6 | -9.45 ± 0.05 | 6 | -9.44 ± 0.03 | 6 | -9.37 ± 0.04 | 6 | -9.56 ± 0.03 | 6 | -9.19 ± 0.05## | 6 |
| Carvedilol | -8.72 ± 0.04 | 6 | -9.06 ± 0.04 | 6 | -9.23 ± 0.05# | 6 | -9.14 ± 0.05 | 6 | -9.09 ± 0.04 | 6 | -8.98 ± 0.02 | 6 |
| CGP 12177 | -8.93 ± 0.04 | 6 | -9.39 ± 0.03 | 6 | -9.45 ± 0.04 | 6 | -9.41 ± 0.05 | 6 | -9.36 ± 0.04 | 6 | -9.19 ± 0.04 | 11 |
| CGP 20712A | -8.51 ± 0.09 | 6 | -8.76 ± 0.12 | 6 | -8.87 ± 0.09 | 6 | -8.81 ± 0.07 | 7 | -8.62 ± 0.08 | 7 | -8.75 ± 0.08 | 8 |
| Cimaterol | -6.21 ± 0.04 | 5 | -6.49 ± 0.06 | 5 | -6.55 ± 0.02 | 5 | -6.50 ± 0.06 | 5 | -6.29 ± 0.06 | 5 | -6.45 ± 0.05 | 6 |
| ICI 118551 | -6.57 ± 0.03 | 6 | -6.92 ± 0.08 | 6 | -6.86 ± 0.03 | 6 | -6.83 ± 0.05 | 6 | -6.75 ± 0.03 | 6 | -6.76 ± 0.04 | 6 |
| Isoprenaline | -5.86 ± 0.11 | 3 | -6.13 ± 0.06 | 3 | -6.38 ± 0.17 | 3 | -6.13 ± 0.07 | 3 | -6.48 ± 0.13 | 3 | -6.07 ± 0.07 | 6 |
| Metoprolol | -7.33 ± 0.03 | 6 | -7.69 ± 0.05 | 6 | -7.59 ± 0.02 | 6 | -7.54 ± 0.04 | 6 | -7.42 ± 0.05 | 6 | -7.59 ± 0.05 | 6 |
| Noradrenaline | -5.36 ± 0.06 | 6 | -5.65 ± 0.07 | 5 | -5.87 ± 0.04# | 6 | -5.78 ± 0.05 | 6 | -6.15 ± 0.07## | 6 | -5.63 ± 0.06 | 6 |
| Pindolol | -8.38 ± 0.11 | 6 | -8.70 ± 0.11 | 6 | -8.72 ± 0.10 | 6 | -8.69 ± 0.12 | 6 | -8.50 ± 0.09 | 6 | -8.57 ± 0.09 | 6 |
| Propranolol | -8.00 ± 0.02 | 10 | -8.31 ± 0.04 | 10 | -8.33 ± 0.03 | 10 | -8.29 ± 0.05 | 10 | -8.08 ± 0.05 | 10 | -8.17 ± 0.03 | 5 |
| Terbutaline | -3.79 ± 0.07 | 5 | -4.01 ± 0.08 | 5 | -4.00 ± 0.07 | 5 | -4.04 ± 0.07 | 5 | -4.13 ± 0.07 | 5 | -3.79 ± 0.02 | 6 |
| Xamoterol | -6.97 ± 0.06 | 7 | -7.19 ± 0.08 | 7 | -7.22 ± 0.08 | 7 | -7.28 ± 0.05 | 7 | -7.17 ± 0.07 | 7 | -7.26 ± 0.05 | 7 |
|  |  |  |  |  |  |  |  |  |  |  |  |  |
| ^3^H-CGP 12177 * | 0.76 ± 0.05 | 10 | 0.32 ± 0.04 | 10 | 0.36 ± 0.04 | 10 | 0.33 ± 0.03 | 10 | 0.34 ± 0.04 | 9 | 0.48 ± 0.03 | 10 |
|  |  |  |  |  |  |  |  |  |  |  |  |  |
| Fmol/mg prot** | 2084 ± 157 | 10 | 219 ± 21 | 10 | 472 ± 39 | 10 | 392 ± 34 | 10 | 499 ± 35 | 9 | 1167 ± 117 | 10 |

Log K_D_ values obtained from ^3^H-CGP 12177 whole cell binding in two additional stable cell lines for each of WT, Gly49 and Arg 389 mutant receptors. Values are mean ± s.e.mean. of n separate determinations.

*The K_D_ value for ^3^H-CGP 121777 in nM determined from saturation binding.

**Receptor expression level given in fmol/mg protein

One-way ANOVA wit post hoc Neuman-Keuls was performed. The affinity at each cell line (V6, V87, W81 and W97) were compared with both WT cell lines. # = p<0.05 and ## = p<0.001 where the value obtained for a polymorphic cell line is significantly different from the value obtained for both the T3 and T88 WT cell lines. NB via this method bucindolol appears statistically significantly different from either of the WT cell lines, even though the value obtained is midway between the values obtained for the WT cell lines.

Table S2

Affinity of β-adrenoceptor ligands for the wildtype and polymorphic variants of the β1-adrenoceptor in transiently transfected populations

|  | WT (transient) | n | Gly49/Gly389 (transient) | n | Ser49/Arg389 (transient) | n |
| --- | --- | --- | --- | --- | --- | --- |
| Adrenaline | -4.68 ± 0.07 | 4 | -4.59 ± 0.05 | 4 | -4.54 ± 0.06 | 4 |
| Bisoprolol | -7.88 ± 0.02 | 4 | -7.78 ± 0.04 | 4 | -7.78 ± 0.06 | 4 |
| Bucindolol | -8.88 ± 0.01 | 4 | -8.80 ± 0.09 | 4 | -8.74 ± 0.06 | 4 |
| Carvedilol | -8.92 ± 0.07 | 4 | -8.83 ± 0.12 | 4 | -8.83 ± 0.08 | 4 |
| CGP 12177 | -9.21 ± 0.02 | 4 | -9.17 ± 0.05 | 4 | -9.10 ± 0.04 | 4 |
| CGP 20712A | -8.76 ± 0.04 | 3 | -8.73 ± 0.05 | 3 | -8.66 ± 0.03 | 3 |
| Noradrenaline | -5.30 ± 0.06 | 4 | -5.24 ± 0.08 | 4 | -5.22 ± 0.09 | 4 |

Log K_D_ values obtained from ^3^H-CGP 12177 whole cell binding in 3 or 4 separate populations of transiently transfected cells for each receptor. Values are mean ± s.e.mean. of n separate determinations. One-way ANOVA with post hoc Neuman-Keuls was performed and there was no statistical difference between the affinity of any ligand for the WT receptor compared with the affinity at the Gly49/Gly389 or Ser49/Arg389 receptor.

Table S3.

Agonist responses occurring via the wildtype and polymorphic variants of the β1-adrenoceptor

|  | cimaterol | % isop | n | CGP 12177 | %isop | n | bisoprolol | % isop | n | nebivolol | % isop | n | xamoterol | % isop | n |
| --- | --- | --- | --- | --- | --- | --- | --- | --- | --- | --- | --- | --- | --- | --- | --- |
| WT cell line T3 |  |  |  | -8.12 ± 0.06 | 55.6 ± 1.3 | 5 | No response | 0 | 5 | -8.95 ± 0.06 | 2.6 ± 0.3 | 5 | -8.25 ± 0.05 | 48.0 ± 2.3 | 5 |
| WT cell line T88 |  |  |  | -7.96 ± 0.02 | 29.4 ± 2.4 | 5 | No response | 0 | 5 | No response | 0 | 5 | -8.19 ± 0.06 | 23.8 ± 2.7 | 5 |
| Transient WT | -8.32 ± 0.03 | 86.4 ± 5.0 | 4 | -8.30 ± 0.07 | 49.7 ± 2.1 | 4 |  |  |  |  |  |  |  |  |  |
| Gly49/Gly389 Cell line V6 |  |  |  | -7.82 ± 0.04 | 40.3 ± 2.2 | 5 | No response | 0 | 5 | -9.65 ± 0.09 | 1.1 ± 0.2 | 5 | -8.09 ± 0.04 | 34.0 ± 1.4 | 5 |
| Gly49/Gly389 Cell line V87 |  |  |  | -7.94 ± 0.03 | 30.2 ± 1.7 | 5 | No response | 0 | 5 | -9.02 ± 0.19 | 1.5 ± 0.3 | 3 | -8.09 ± 0.07 | 27.1 ± 1.9 | 5 |
| Transient Gly49/Gly389 | -8.29 ± 0.13 | 77.7 ± 3.8 | 3 | -8.17 ± 0.03 | 46.3 ± 3.9 | 3 |  |  |  |  |  |  |  |  |  |
| Ser49/Arg389 Cell line W81 |  |  |  | -7.03 ± 0.07 | 16.6 ± 1.0 | 4 | No response | 0 | 5 | -9.18 ± 0.20 | 1.8 ± 0.5 | 5 | -7.80 ± 0.02 | 35.2 ± 2.4 | 5 |
| Ser49/Arg389 Cell line W93 |  |  |  | -7.75 ± 0.05 | 52.6 ± 2.4 | 5 | No response | 0 | 5 | -9.43 ± 0.08 | 1.1 ± 0.1 | 5 | -8.06 ± 0.03 | 37.2 ± 1.0 | 5 |
| Transient Ser49/Arg389 | -8.24 ± 0.04 | 84.5 ± 4.4 | 4 | -8.08 ± 0.07 | 40.9 ± 3.8 | 4 |  |  |  |  |  |  |  |  |  |

Log EC_50_ values for ligands in two additional stable cell lines for each of WT, Gly49 and Arg 389 mutant receptors of the human β1-adrenoceptor and in 3 or 4 separate populations of transiently transfected cells for each receptor. Values are mean ± s.e.mean. of n separate determinations.

Table S4.

Agonist responses for ligands with biphasic responses at the wildtype and polymorphic variants of the β1-adrenoceptor in two additional stable cell lines for each receptor and transiently transfected cells.

|  | Log EC_50_ Site 1 | Log EC_50_ Site 2 | % Site 1 | % isoprenaline | n |
| --- | --- | --- | --- | --- | --- |
| Pindolol |  |  |  |  |  |
| WT cell line T3 | -8.88 ± 0.20 | -5.96 ± 0.11 | 40.8 ± 2.9 | 33.2 ± 4.2 | 5 |
| WT cell line T88 | -9.16 ± 0.13 | -6.36 ± 0.13 | 38.1 ± 3.5 | 13.1 ± 1.2 | 5 |
| Transient transfection WT | -9.13 ± 0.11 | -6.21 ± 0.21 | 56.5 ± 3.6 | 28.7 ± 1.5 | 4 |
| Gly49/Gly389 Cell line V6 | -8.91 ± 0.19 | -5.63 ± 0.07 | 38.7 ± 2.2 | 17.1 ± 1.2 | 5 |
| Gly49/Gly389 Cell line V87 | -9.07 ± 0.14 | -5.74 ± 0.13 | 41.7 ± 1.0 | 16.0 ± 1.2 | 4 |
| Transient transfection Gly49/Gly389 | -8.75 ± 0.14 | -6.12 ± 0.26 | 48.4 ± 1.3 | 26.3 ± 3.0 | 3 |
| Ser49/Arg389 Cell line W81 | -8.96 ± 0.07 | -6.81 ± 0.14 | 47.1 ± 2.8 | 8.0 ± 0.3 | 4 |
| Ser49/Arg389 Cell line W93 | -8.76 ± 0.05 | -6.15 ± 0.11 | 40.7 ± 1.8 | 16.5 ± 1.4 | 5 |
| Transient transfection Ser49/Arg389 | -9.01 ± 0.08 | -6.08 ± 0.24 | 64.5 ± 5.1 | 23.9 ± 2.4 | 4 |
|  |  |  |  |  |  |
| Bucindolol |  |  |  |  |  |
| WT cell line T3 | -9.15 ± 0.09 | -7.09 ± 0.15 | 75.6 ± 4.5 | 43.4 ± 2.0 | 4 |
| WT cell line T88 | -9.63 ± 0.07 | -7.46 ± 0.09 | 48.0 ± 4.2 | 23.6 ± 2.4 | 5 |
| Gly49/Gly389 Cell line V6 | -9.55 ± 0.08 | -7.45 ± 0.10 | 54.0 ± 2.7 | 27.5 ± 1.0 | 5 |
| Gly49/Gly389 Cell line V87 | -9.51 ± 0.09 | -7.34 ± 0.23 | 69.3 ± 7.5 | 22.8 ± 1.1 | 4 |
| Ser49/Arg389 Cell line W81 | -9.14 ± 0.08 | -6.22 ± 0.15 | 47.6 ± 3.3 | 34.8 ± 2.0 | 4 |
| Ser49/Arg389 Cell line W93 | -9.23 ± 0.12 | -7.50 ± 0.07 | 51.5 ± 4.0 | 32.8 ± 1.1 | 5 |
|  |  |  |  |  |  |
| Carvedilol |  |  |  |  |  |
| WT cell line T3 | -8.76 ± 0.08 | -7.19 ± 0.09 | 56.0 ± 4.2 | 10.5 ± 1.1 | 9 |
| WT cell line T88 | Response too small to determine each site | | | 3.4 ± 0.9 | 9 |
| Gly49/Gly389 Cell line V6 | -9.34 ± 0.04 | -6.99 ± 0.04 | 55.3 ± 4.0 | 5.3 ± 0.5 | 7 |
| Gly49/Gly389 Cell line V87 | -9.55 ± 0.11 | -7.55 ± 0.16 | 50.7 ± 3.2 | 7.0 ± 0.8 | 8 |
| Ser49/Arg389 Cell line W81 | -9.43 ± 0.08 | -6.92 ± 0.16 | 49.7 ± 3.3 | 3.4 ± 0.1 | 8 |
| Ser49/Arg389 Cell line W93 | -9.22 ± 0.07 | -7.12 ± 0.09 | 51.5 ± 3.0 | 5.7 ± 0.5 | 8 |

Log EC_50_ values for ligands having a biphasic concentration response curve in two additional stable cell lines for each of WT, Gly49 and Arg 389 mutant receptors of the human β1-adrenoceptor, and transiently transfected populations of cells for each receptor. The % of the response occurring at site 1 and % maximum isoprenaline response for the overall response is also given. Values are mean ± s.e.mean. of n separate determinations.

Table S5.

Relationship between receptor expression level and efficacy of partial agonists at the two agonist conformations of the wildtype and polymorphic variants of the β1-adrenoceptor

|  |  |  |  | Catecholamine conformation | |  | Secondary conformation | |
| --- | --- | --- | --- | --- | --- | --- | --- | --- |
|  | Expression level (fmol/mg protein) | n |  | Xamoterol % isop max | n |  | CGP 12177 % isop max | n |
| WT T88 | 219 ± 21 | 10 |  | 23.8 ± 2.7 | 5 |  | 29.4 ± 2.4 | 5 |
| WT original | 611 ± 98 | 11 |  | 28.0 ± 2.4 | 7 |  | 34.8 ± 2.7 | 13 |
| WT T3 | 2084 ± 157 | 10 |  | 48.0 ± 2.3 | 5 |  | 55.6 ± 1.3 | 5 |
|  |  |  |  |  |  |  |  |  |
| Gly49/Gly389 V87 | 392 ± 34 | 10 |  | 27.1 ± 1.9 | 5 |  | 30.2 ± 1.7 | 5 |
| Gly49/Gly389 V6 | 472 ± 39 | 10 |  | 34.0 ± 1.4 | 5 |  | 40.3 ± 2.2 | 5 |
| Gly49/Gly389 original | 819 ± 75 | 10 |  | 29.4 ± 4.5 | 9 |  | 36.0 ± 1.3 | 12 |
|  |  |  |  |  |  |  |  |  |
| Ser49/Arg389 W81 | 499 ± 35 | 9 |  | 35.2 ± 2.4 | 5 |  | 16.6 ± 1.0 | 4 |
| Ser49/Arg389 original | 1068 ± 142 | 9 |  | 52.1 ± 4.5 | 9 |  | 59.1 ± 1.6 | 13 |
| Ser49/Arg389 W93 | 1167 ± 117 | 10 |  | 37.2 ± 1.0 | 5 |  | 52.6 ± 2.4 | 5 |

Receptor expression levels and ligand efficacy expressed as % isoprenaline maximum response for a catecholamine conformation partial agonist (xamoterol) and a secondary conformation agonist (CGP 12177).

**Figure S1**

**^3^H-inositol phosphate accumulation in cells expressing the wildtype β1-adrenoceptor and polymorphic variants.**

^3^H-inositol phosphate accumulation in A wildtype cells, B Gly49/Gly389 cells and C Ser49/Arg389 cells. Bars are mean ± s.e.mean of triplicate determinations. These single experiments are representative of 4 separate experiments in each case and demonstrate a lack of Gq-coupled inositol phosphate accumulation in response to β-adrenoceptor ligands.

**Figure S2**

**MAPKinase activation of the wildtype β1-adrenoceptor and polymorphic variants.**

MAPKinase activation in A wildtype cells, B Gly49/Gly389 cells and C Ser49/Arg389 cells. Bars are mean ± s.e.mean of triplicate determinations. These single experiments are representative of 3 separate experiments in each case and demonstrate a lack of ERK1/2 MAPKinase stimulation by β-adrenoceptor ligands.

**Figure S3**

Correlation plot and statistical analysis for the affinity of all the ligands from Supplementary Table 1 for the WT (clone T88 x-axis) and WT (clone T3), polymorphic variants, and transient populations (y-axis). The linear regression lines are not shown on the graph to ensure that the symbols are still visible. This shows that the affinity of ligands for the different β1-adrenoceptor variants is similar to that for the wildtype receptor.
